# Is the coverage of google scholar enough to be used alone for systematic reviews

**DOI:** 10.1186/1472-6947-13-7

**Published:** 2013-01-09

**Authors:** Jean-François Gehanno, Laetitia Rollin, Stefan Darmoni

**Affiliations:** 1 Institute of Occupational Health, Rouen University Hospital and University of Rouen, 1 rue de Germont, 76000, Rouen, France; 2 CISMeF-TIBS-LITIS EA 4108, Rouen University Hospital, Rouen, France

**Keywords:** Bibliometrics, Google scholar, Information retrieval methods, Systematic reviews

## Abstract

**Background:**

In searches for clinical trials and systematic reviews, it is said that Google Scholar (GS) should never be used in isolation, but in addition to PubMed, Cochrane, and other trusted sources of information. We therefore performed a study to assess the coverage of GS specifically for the studies included in systematic reviews and evaluate if GS was sensitive enough to be used alone for systematic reviews.

**Methods:**

All the original studies included in 29 systematic reviews published in the Cochrane Database Syst Rev or in the JAMA in 2009 were gathered in a gold standard database. GS was searched for all these studies one by one to assess the percentage of studies which could have been identified by searching only GS.

**Results:**

All the 738 original studies included in the gold standard database were retrieved in GS (100%).

**Conclusion:**

The coverage of GS for the studies included in the systematic reviews is 100%. If the authors of the 29 systematic reviews had used only GS, no reference would have been missed. With some improvement in the research options, to increase its precision, GS could become the leading bibliographic database in medicine and could be used alone for systematic reviews.

## Background

The release of the beta version of Google Scholar (GS) (http://scholar.google.com) in November 2004 generated much media coverage and academic commentary. It has been met with both enthusiasm and criticism but Google and GS now lead more visitors to many biomedical journal websites than does Medline via its PubMed interface [1-3].

GS searches retrieve results that include scholarly literature citations as well as peer-reviewed publications, theses, books, abstracts, and other articles from academic publishers, professional organizations, and preprint repositories, universities, and other scholarly organizations. Therefore, GS is able to retrieve more types of literature compared with medical literature database retrieval search engines, like PubMed [4]. GS is also able to identify some of the references of PubMed, but not all [5].

Doctors are encouraged to consult GS for browsing and serendipitous discovery, not for literature reviews [1]. In searches for clinical trials and systematic reviews, it is said that GS should never be used in isolation, but in addition to PubMed, Cochrane, and other trusted sources of information [1]. Many studies have demonstrated that a single search engine does not capture all of the available articles, and using two or more databases provides greater coverage of all possible citations [6-17].

Nevertheless, the coverage of GS is increasing and, despite the fact that it is said to be not exhaustive, is it exhaustive enough for the studies that are considered of enough quality or relevance for systematic reviews [18].

Therefore, the objective of this study was to assess the coverage of GS, and its potential recall, specifically for such studies, and therefore to assess if this database could be used alone for systematic reviews.

## Methods

The first step aimed at identifying a subset of studies selected by experts to be included in systematic reviews. We searched Medline in December 2009 for the systematic reviews published in the JAMA or the Cochrane Library. For the JAMA, we used the most specific search string proposed by Montori et al., with limits for the years 2008 and 2009 [19]. For the Cochrane Library, we examined all the systematic reviews published in the Cochrane Database Syst Rev. 2009 Jul 8;(3).

We excluded the systematic reviews using less than 2 bibliographic databases in their search and those which restricted the search to English language studies.

The gold standard database was then built by gathering all the studies included in the systematic reviews we selected, excluding abstracts and personal communications. We considered Gray literature (*i.e.* written material that is not published commercially or is not generally accessible) as a specific subset, but we included these references in the gold standard database.

GS was searched for each reference, one by one, by searching with the title of each of the studies included in the gold standard database. Recall (*i.e.* the proportion of studies retrieved from the database) of GS were computed for each review published in the Cochrane Library or the JAMA.

## Results

Overall, 14 reviews from the Cochrane library and 15 reviews from the JAMA were included. To identify all the possible relevant studies, each systematic review from the Cochrane Library and from the JAMA had searched between 3 and 10 (mean: 5.4) and between 2 and 9 (mean : 4) different databases, respectively. All of them searched Medline and 17 mentioned to have also scanned the reference list of the studies they included.

The 29 systematic reviews had included 755 original studies. Among them, 733 were published in peer-reviewed journals and 5 were detailed only in document belonging to the gray literature. The 18 remaining studies were referenced only as an abstract or as personal communication and were therefore not included in the gold standard database, which included finally 738 original studies. All the 738 studies were identified in GS, leading to 100% coverage.

The detailed results are presented in Table 1.

**Table 1:** Recall of Google scholar for the 29 systematic reviews

**Source of the systematic review**	**Title of the systematic review**	**Number of databases searched by the authors**	**Number of studies included in the review**	**Number of studies found in Google Scholar**
Cochrane Library	Antidepressants versus placebo for depression in primary care	8	14	14
Cochrane Library	Artemisinin-based combination therapy for treating uncomplicated malaria	6	49	49
Cochrane Library	Brief interventions for heavy alcohol users admitted to general hospital wards	5	11	11
Cochrane Library	Combined DTP-HBV-HIB vaccine versus separately administered DTP-HBV and HIB vaccines for primary prevention of diphtheria, tetanus, pertussis, hepatitis B and Haemophilusinfluenzae B (HIB)	3	18	18
Cochrane Library	Erythropoietin or Darbepoetin for patients with cancer--meta-analysis based on individual patient data	3	39	39
Cochrane Library	Green tea (Camellia sinensis) for the prevention of cancer	7	51	51
Cochrane Library	Incentive spirometry for prevention of postoperative pulmonary complications in upper abdominal surgery	5	11	11
Cochrane Library	Interventions to prevent occupational noise induced hearing loss	10	20	20
Cochrane Library	Non-pharmacological interventions for assisting the induction of anaesthesia in children	7	17	17
Cochrane Library	Oral iron supplementation for preventing or treating anaemia among children in malaria-endemic areas	5	68	68
Cochrane Library	Pharmacotherapy for anxiety disorders in children and adolescents	4	25	25
Cochrane Library	Single dose oral flurbiprofen for acute postoperative pain in adults	4	11	11
Cochrane Library	The effects of antimicrobial therapy on bacterial vaginosis in non-pregnant women	5	24	24
Cochrane Library	Therapeutic interventions for symptomatic treatment in Huntington’s disease	4	20	20
JAMA	Acute-onset floaters and flashes: is this patient at risk for retinal detachment?	2	17	17
JAMA	Adiponectin levels and risk of type 2 diabetes: a systematic review and meta-analysis	3	14	14
JAMA	Allogeneic stem cell transplantation for acute myeloid leukemia in first complete remission: systematic review and meta-analysis of prospective clinical trials	3	17	17
JAMA	Aspirin for the prevention of cardiovascular events in patients with peripheral artery disease: a meta-analysis of randomized trials.	4	15	15
JAMA	Bed bugs (Cimexlectularius) and clinical consequences of their bites.	2	49	49
JAMA	Cancer survivors and unemployment: a meta-analysis and meta-regression.	5	24	24
JAMA	Cardiorespiratory fitness as a quantitative predictor of all-cause mortality and cardiovascular events in healthy men and women: a meta-analysis.	2	32	32
JAMA	Combined corticosteroid and antiviral treatment for Bell palsy: a systematic review and meta-analysis.	6	17	17
JAMA	Corticosteroids in the treatment of severe sepsis and septic shock in adults: a systematic review	4	19	19
JAMA	Diagnostic performance of computed tomography angiography in peripheral arterial disease: a systematic review and meta-analysis	3	20	20
JAMA	Interaction between the serotonin transporter gene (5-HTTLPR), stressful life events, and risk of depression: a meta-analysis.	3	14	14
JAMA	Lipoprotein(a) concentration and the risk of coronary heart disease, stroke, and nonvascular mortality.	2	36	36
JAMA	Predictive value of factor V Leiden and Prothrombin G20210A in adults with venous thromboembolism and in family members of those with a mutation. A systematic review	5	46	46
JAMA	Sexual abuse and lifetime diagnosis of somatic disorders: a systematic review and meta-analysis	9	22	22
JAMA	Treatment of fibromyalgia syndrome with antidepressants: a meta-analysis.	6	18	18
**Total**			**738**	**738 (100%)**

As a side result, we discovered that a striking number of bibliographic references included major errors, *i.e.* errors that involve the data elements by which references are searched by users in Medline [20]. Overall, 10 references contained at least one major error, some of them containing up to 3 major errors.

Some of the reviews concentrated these citation errors. For example, among the 24 references included in the Cochrane review " The effects of antimicrobial therapy on bacterial vaginosis in non-pregnant women", 5 contained at least one major error.

## Discussion

Performing systematic reviews is a complex and time consuming task, because of the body of literature to be searched and the high number of databases that must be used, considering that no one of them is considered exhaustive. The use of GS is increasing, as well as its coverage, and we wanted to assess if this coverage is high enough to be used alone in systematic reviews.

GS allowed to retrieve 100% of the studies included in the systematic reviews we studied, and which covered many different fields of medicine.

Although GS does not cover all the medical literature, we therefore observed that its coverage of the studies of sufficient quality or relevance to be included in a systematic review was complete. In other words, if the authors of these 29 systematic reviews had used only GS, they would have obtained the very same results.

The validity of our gold standard database could nevertheless be questioned. To identify the studies that worth to be included in a systematic review, we relied on the works of the experts used as reviewer in the systematic reviews we included, since all of them used at least 2 independent reviewers. Furthermore, we excluded from our gold standard database personal communications, because they cannot be retrieved by any database, and abstracts because it has been clearly demonstrated that such abstracts often display non-valid results [21,22]. Considering the methods used by the authors of the systematic reviews we selected, the use of at least two independent reviewers to select relevant articles in these reviews, the high number of databases searched and the absence of restriction to English studies in each of them, we can also assume that, for each topic covered, all the relevant studies were identified. Therefore, we can assume that our gold-standard database really included all the studies of sufficient quality and relevant to the topics covered by the systematic reviews, and only them.

We chose to study the systematic reviews published by the JAMA and Cochrane because they usually don’t restrict their search to English literature and they use more than one database to perform the search, which is not the case of most of the systematic reviews published by the Annals of Internal Medicine, for example.

Although the recall of GS was 100%, the amount of information delivered by GS was heterogeneous. Yet, some of the studies were only identified as "citations", which means that GS only displayed the authors, the title of the article and the name, year and pages of the journals. This can be considered as insufficient, but traditional biomedical databases (such as Medline or Embase) do the same for old articles or for articles published in another language that English. Furthermore, this is exactly the same situation when authors of systematic reviews perform hand searching in the reference list of selected articles. Therefore, we considered valid to include these hits as positive results.

This 100% coverage of GS can be seen as amazing, since no single database is supposed to be exhaustive, even for good quality studies. For example, the recall ratios of Medline for randomized control trials (RCTs) only stand between 35% and 56% [23,24]. Since GS accesses only 1 million of the some 15 million records at PubMed, how can our results be explained? In fact, through agreements with publishers, GS accesses the “invisible” or “deep” Web, that is, commercial Web sites the automated “spiders” used by search engines such as Google cannot access. Furthermore, we observed in our study that most of the articles indentified by GS were found directly on the publishing journal web-sites, and not on the PubMed web-site.

Nevertheless, while its advantages are substantial, GS is not without flaws. The shortcomings of the system and its search interface have been well documented in the literature and include lack of reliable advanced search functions (*e.g*. no MeSH term subheading search function), lack of controlled vocabulary, lack of a “similar pages” feature, and issues regarding scope of coverage and currency [4,5,25]. Furthermore, whereas PubMed displays results in a chronological order, GS places more relevance on articles that are cited most often. Therefore, the citations located are reportedly biased toward older literature [26,27]. This last point can also be viewed as an advantage, since it allows to identify quickly landmark articles, *i.e.* articles of importance in a field. Yet, when comparing searches with PubMed and Google Scholar by evaluating the first 20 articles recovered for four clinical questions for relevance and quality, Nourbakhsh and coll. demonstrated that GS provided more relevant results that PubMed, although the difference was not significant (p=0.116) [28].

GS has been reported to be less precise than PubMed, since it retrieves hundreds or thousands of documents, most of them being irrelevant [29,30]. Nevertheless, we should not overestimate the precision of PubMed in real life since Precision and recall of a search in a database is highly dependent on the skills of the user [10]. Many of them overestimate the quality of their searching performance, and experienced reference librarians typically retrieve about twice as many citations as do less experienced users [31,32].

Although this was not the purpose of our study, we tried to assess the precision of GS for some of the clinical questions that were studied by the systematic reviews.

For example, searching for "(Erythropoietin or Darbepoetin) cancer" in GS gave a recall of 100% and a precision of 0.1% (36,630 articles found, for 36 included in the systematic review). In GS, the search string "(depression treatment placebo antidepressant) ("general practice" OR "Primary care")" identified 16100 articles, leading to a recall of 100% and a precision of 0.09 (14 articles included in the corresponding systematic review).

## Conclusion

In conclusion, the coverage of GS is much higher than previously thought for high quality studies. GS is highly sensitive, easy to search and could be the first choice for systematic reviews or meta-analysis. It could even be used alone. It just requires some improvement in the advanced search features to improve its precision and to become the leading bibliographic database in medicine.

## Competing interests

The authors declare they have no competing interest.

## Authors’ contribution

JFG conceived of the study. JFG and LR collected the data. JFG, LR and SJD analyzed the data and drafted the manuscript. All authors read and approved the final manuscript.

## Pre-publication history

The pre-publication history for this paper can be accessed here:

http://www.biomedcentral.com/1472-6947/13/7/prepub
